# Ragas: integration and enhanced visualization for single cell subcluster analysis

**DOI:** 10.1093/bioinformatics/btae366

**Published:** 2024-06-12

**Authors:** Uthra Balaji, Juan Rodríguez-Alcázar, Preetha Balasubramanian, Cynthia Smitherman, Jeanine Baisch, Virginia Pascual, Jinghua Gu

**Affiliations:** Drukier Institute for Children’s Health and Department of Pediatrics, Weill Cornell Medicine, New York, NY 10021, United States; Drukier Institute for Children’s Health and Department of Pediatrics, Weill Cornell Medicine, New York, NY 10021, United States; Drukier Institute for Children’s Health and Department of Pediatrics, Weill Cornell Medicine, New York, NY 10021, United States; Drukier Institute for Children’s Health and Department of Pediatrics, Weill Cornell Medicine, New York, NY 10021, United States; Drukier Institute for Children’s Health and Department of Pediatrics, Weill Cornell Medicine, New York, NY 10021, United States; Drukier Institute for Children’s Health and Department of Pediatrics, Weill Cornell Medicine, New York, NY 10021, United States; Drukier Institute for Children’s Health and Department of Pediatrics, Weill Cornell Medicine, New York, NY 10021, United States

## Abstract

**Summary:**

Subcluster analysis is a powerful means to improve clustering and characterization of single cell RNA-Seq data. However, there are no existing tools to systematically integrate results from multiple subclusters, which creates hurdles for accurate data quantification, visualization, and interpretation in downstream analysis. To address this issue, we developed Ragas, an R package that integrates multi-level subclustering objects for streamlined analysis and visualization. A new data structure was implemented to seamlessly connect and assemble miscellaneous single cell analyses from different levels of subclustering, along with several new or enhanced visualization functions. Moreover, a re-projection algorithm was developed to integrate nearest-neighbor graphs from multiple subclusters in order to maximize their separability on the combined cell embeddings, which significantly improved the presentation of rare and homogeneous subpopulations.

**Availability and implementation:**

The Ragas package and its documentation can be accessed through https://github.com/jig4003/Ragas and its source code is also available at https://zenodo.org/records/11244921.

## 1 Introduction

Recent developments in computational tools have provided critical support to investigate transcriptional and phenotypical changes from massive single cell RNA-Seq data. Several frameworks have been developed, including Seurat (Hao *et al.* 2021), Scanpy (Wolf *et al.* 2018), and SingleCellExperiment (Amezquita *et al.* 2020), which provide convenient interfaces to orchestrate critical steps in the single-cell analysis workflow, from data processing, clustering, to differential analysis and visualization.

One central challenge of single cell data analysis is to accurately cluster hundreds of thousands of cells into their corresponding cell types based on gene expression, which is often accomplished using a procedure called guided clustering (Stuart *et al.* 2019). Although a one–off guided clustering is often deemed effective in analyzing main cell populations from complex cell mixtures, such as from whole tissues or peripheral blood mononuclear cells (PBMCs), its efficacy to characterize rare or homogeneous subpopulations is very limited.

A simple remedy to improve clustering and annotation of rare or homogeneous subpopulations is to perform subcluster analysis (Luecken and Theis 2019, Nehar-Belaid *et al.* 2020) by first dividing total single cells into several main clusters that each contains cells from one major cell type, followed by looping through the main clusters and re-cluster cells for refined subpopulations. However, subcluster analysis is often performed in an *ad hoc* fashion to improve the accuracy and resolution of single cell analysis yet there are no existing tools to integrate data from different subclusters. Therefore, critical gaps remain in the analysis and visualization of subpopulations of interest. One such example is the lack of effective methods to embed subcluster structures back into the total cells so that subpopulations from distinct compartments can be jointly analyzed and visualized. Indeed, many existing studies (Zhang *et al.* 2020, Guo *et al.* 2022, Zheng *et al.* 2022) analyzed subclusters separately from one another yet overlooked the hierarchy of cell subsets, which may lead to partial or skewed interpretation of essential results, such as that from differential cell proportion analysis.

In this work, we introduce Ragas (R Advanced Gallery for Analysis of Subclusters), an R-based framework for integrative analysis and visualization of single-cell subcluster data. Ragas offers three major contributions: (i) A unified data structure that is compatible with the Seurat object is defined to coordinate analysis and visualization of single cell data. (ii) A re-projection algorithm is developed to combine cell embeddings from multiple subclusters, which can display sufficient structural granularities for rare or homogeneous subpopulations. (iii) As an addition to existing Seurat visualization functions, Ragas provides an intuitive interface to generate new or enhanced single cell plots (Supplementary Results and Supplementary Table S1). Ragas is also designed to be compatible with many popular scRNA-Seq workflows, which gives users great flexibility to choose among different pre-processing and analysis methods, such as for batch integration and clustering. Using a pediatric systemic lupus erythematosus (SLE) dataset as an example, we showcased the ease-of-use, optimized data representation and visualization by Ragas that led to critical biological insights.

## 2 Software implementation

**Figure 1.**
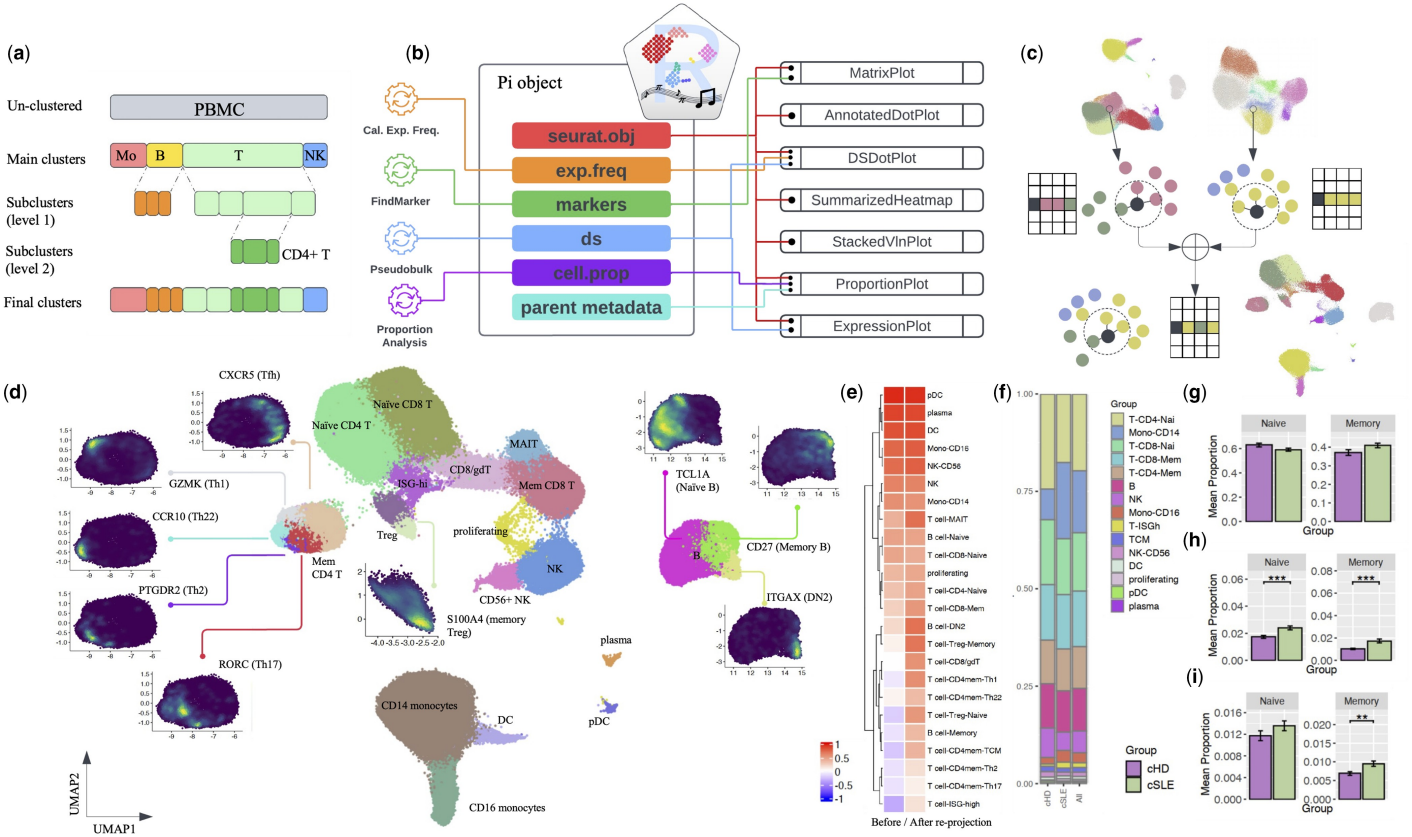
Integrative subcluster analysis using Ragas. (a) Example schematic of 2-level subcluster analysis of PBMC data. In the main cluster analysis, total PBMCs are clustered into four major immune cell populations: monocytes (Mo), B cells, T cells, and natural killer cells (NK). The first subcluster analysis re-clustered B and T cells; the second subcluster analysis further subset and re-clustered CD4+ T cells. (b) A diagram showing components of a Pi object and their connections to various plotting functions. (c) Illustration of the subcluster re-projection algorithm by combining nearest neighbor graphs. (d) A re-projected UMAP at the PBMC level showing marker expression for subpopulations of B cells, Tregs, and memory CD4+ cells. (e) Heatmap showing the average silhouette width of the subcluster labels on UMAPs from before and after re-projection. (f) Bar plot showing immune cell composition of PBMCs from the pediatric SLE dataset. (g–i) Differential cell proportion analysis conditioned on different reference populations, including total Tregs (g), total T cells (h), and total PBMCs (i).

Ragas is developed to facilitate streamlined data integration and visualization for “multi-level” subclustering (Fig. 1a). In such analytical design, initial clustering is performed at the total cell level (e.g. PBMCs), generating the first round of clusters that we refer to as “main clusters.” The main clusters typically reflect major cell populations but may have insufficient resolution or accuracy for subpopulations. When deemed necessary, cells from one or more main clusters (e.g. T cells) can be subsetted to perform further subcluster analysis, generating data that we refer to as the “child” of the main cluster object (i.e. parent). In some cases, one round of subcluster analysis may not be adequate to capture the heterogeneity within a complex cell mixture, especially for rare or homogeneous subpopulations (e.g. T helper subsets), hence requiring additional levels of subclustering.

### 2.1 The post-integration object orchestrates scRNA-Seq analysis and visualization

To run Ragas, a Seurat object is required as input, which contains processed data that are filtered and normalized, batch integrated, and clustered. To provide an integrated framework that seamless connects essential components of single-cell RNA-Seq analysis, we introduce a data structure called “post-integration” (Pi) class (Fig. 1b). An object of class Pi contains a Seurat object along with other analytical data, including per-gene expression frequency (“exp.freq”), markers for each cluster (“markers”), differential state analysis results using the pseudobulk approach (“ds”) (Robinson *et al.* 2010, Crowell *et al.* 2020), cell proportion analysis (“cell.prop”), and metadata from the parent Seurat object. The Pi data structure offers convenience in performing scRNA-seq analysis for multi-sample, multi-group study designs, such as differential state analysis and cell proportion analysis to identify genes or cell subsets that show quantitative differences between groups. In line with subcluster analysis, it is worth noting that Ragas also allows cell proportion analysis to be conditioned on different cell compartments so that frequencies of subpopulations can be flexibly calculated and compared within a total population of users’ choice. This can simply be accomplished by linking a child subcluster object (e.g. CD4+ memory T cell) to a specific parent object (e.g. CD4+ T cells, total T cells, or PBMCs) so relative cell proportions can be quantified accordingly.

To complement existing plotting functions from Seurat, some of the popular single-cell plots have been re-developed to provide more versatile layout and flexible display of metadata, such as annotated dot plot, summarized heatmap, and stacked violin plot. Moreover, we have also developed several new functions to help visualize important analysis in the Pi object, including matrix plot to show expression of top markers per cluster, dot plot and expression plot to visualize differential state analysis, and also plots for differential cell proportion analysis.

### 2.2 Subcluster re-projection

To maximize visualizing the granularity of subcluster structures, a re-projection algorithm is implemented to integrate UMAPs from multi-level subcluster analysis (Fig. 1c). Briefly, the corresponding *K* nearest neighbor (KNN) objects for all subclusters (e.g. B and T cells) and their parent object (e.g. PBMCs) are first calculated. KNN distances of the subclusters are then scaled so the average distances among cells within a subcluster align with that of the parent cluster. A weight parameter *w* (0 ≤ *w ≤* 1) is introduced to control the degree to preserve the main cluster structure from the parent object when combining its KNN with KNNs from its child subclusters. For each cell in the parent KNN, *K*_1_ = w*K of the nearest neighbors that are from a different cluster are retained, which will be further merged with the *K−K*_1_ nearest neighbors from the subcluster object to generate the merged neighbor list. The default value for *w* is 1, which typically provides robust re-projections that balance the local subcluster separability and global connectivity. Users are encouraged to explore the effect of smaller *w* when better separability among subclusters are desired (Supplementary Results and Supplementary Figs S1–S3). The new merged neighbor list will then be re-ordered based on their distances. Finally, a new UMAP is generated using the new merged KNN as input, which integrates neighborhood structures from both the parent clusters and subclusters. For cells that do not undergo subclustering analysis, their original nearest neighbors and corresponding distances from the parent data will be preserved.

## 3 Results

To demonstrate the utility and advantages of Ragas for subclustering analysis, we re-analyzed PBMCs from a pediatric SLE dataset (Nehar-Belaid *et al.* 2020) with 33 SLE samples and 11 matched healthy controls (Supplementary Methods). The total PBMCs were first clustered into 15 subclusters, which accurately represented the major immune populations from the myeloid (classical and non-classical monocytes, DC, pDC) and lymphoid lineages (B cell, T cell, natural killer, plasma cell) (Supplementary Figs S4 and S5). However, subpopulations of immune cells were poorly characterized at the total PBMC level, leading to insufficient segregation or even mixtures of immune subsets (Supplementary Figs S6 and S8). To improve the identification of immune subpopulations, we performed multi-level subclustering analysis on the total PBMCs: we first subset and re-clustered B cells and T cells, followed by a second round of subclustering of Treg cells and memory CD4+ T helper cells from the total T cells.

### 3.1 Re-projection untangles B and T cell subsets in PBMC

After subclustering, we identified three B cell subsets (naïve, memory, and DN2 Jenks *et al.* 2018) and 13 T cell subsets (Supplementary Fig. S9), including CD8+ naïve and memory T cells, mucosal-associated invariant T (MAIT) cells, CD8/gdT, naïve and memory regulatory T cells (Tregs), CD4+ central memory T (TCM) cells, helper T cells (Th1, Th2, Th17, Th22), and a T cell subcluster expressing high levels of interferon-stimulated genes (ISGs). However, some of these immune subpopulations, especially CD4+ T helper cells and memory/DN2 B cells, largely overlapped with one another on the original UMAP, making it difficult for users to inspect them at the PBMC level (Supplementary Figs S6 and S10).

Figure 1d shows the re-projected UMAP of PBMCs with B and T cell subclusters integrated. On one hand, the re-projection algorithm nicely preserved the shape and relative locations on the new UMAP for myeloid (monocytes and DCs) and plasma cells that were excluded from subclustering. On the other hand, for T and B cells, structures of the subclusters were successfully integrated into the combined PBMC embedding, with their markers distinctly coloring the corresponding subsets for naive B cells (TCL1A), memory B cells (CD27), DN2 B cells (FCRL5), naïve (CCR7) and memory (S100A4) Tregs, Th1 (GZMK), Th2 (PTGDR2), Th17 (RORC), Th22 (CCR10), and TCMs that express blood T follicular helper (Tfh) marker (CXCR5). Moreover, while detailing the local structures of T and B cell subpopulations, the re-projected UMAP maintained the boundaries between major immune clusters, such as that between T and NK cells, leaving the global structure at the PBMC level intact.

To quantitatively evaluate the advantage of the re-projection method, we calculated the silhouette width, a measure of clustering quality that ranges from −1 (poor) to 1 (good), for the final subcluster labels on UMAP embeddings before and after re-projection (Supplementary Methods). Figure 1e shows that the average silhouette width of any cluster without subclustering from the re-projected UMAP was very similar compared to that from the original PBMC UMAP. However, for cells undergone subcluster analysis, embeddings from the re-projection consistently yielded higher values of silhouette width than the original cell embeddings, validating its robustness and improved resolution to visualize both main and subcluster structures.

### 3.2 Conditional association of Treg subset frequencies with SLE

Frequencies of different cell populations are often compared between biological groups in single-cell RNA-Seq. Depending on the biological hypothesis and complexity of the target cell population, calculation of relative cell proportions is conditioned on a “reference” population, which can either be the total cells (e.g. PBMC), or a specific cell compartment of interest (e.g. CD8+ T cells). To accommodate varying needs in cell proportion analysis, two types of plots, namely “pooled” and “unpooled” proportion plots, are developed in Ragas. While the “pooled” proportion plot is a simple method to visualize cell frequency changes for general purpose (Fig. 1f), the “unpooled” plot is particularly suitable for multi-sample, multi-group study designs. In other words, for unpooled plots, cell counts are summarized (e.g. by sample) and their relative proportions are calculate based on the reference population of choice so that they can be compared and tested between different groups. Figure 1g–i shows the Treg frequency changes between SLE and healthy controls with respect to total Tregs, total T cells, or PBMCs. When comparing within Treg cells, no significant differences in cell frequency were detected between SLE and healthy for naïve or memory Tregs. However, when investigating Treg proportions with respect to the total number of T cells, a significant increase of both naïve and memory Tregs were observed in SLE. A similar expansion of Treg proportions in total PBMC were observed, but only the memory subset achieved statistical significance, not the naïve one. The difference between relative Treg proportions calculated based on total T cells and PBMCs can be linked to a global reduction of T lymphocytes in SLE known as lymphopenia (Supplementary Fig. S11), a complicated process involving a number of pathophysiological factors, including antilymphocyte antibodies and abnormal apoptosis, among others (Fayyaz *et al.* 2015).

## Supplementary Material

btae366_Supplementary_Data
