# Supplementary material for: Ragas: integration and enhanced visualization for single cell subcluster analysis
Source: Bioinformatics. 2024 Jun 12;40(6):btae366. doi: 10.1093/bioinformatics/btae366 (PMC11209553; doi:10.1093/bioinformatics/btae366)
Supplement: btae366_Supplementary_Data [file btae366_supplementary_data.pdf]

Supplementary Data

Ragas: integration and enhanced visualization for single cell subcluster analysis

Supplementary Results 2
Comparing Ragas with existing subclustering integration methods 2
The re-projection weight parameter w 3
Supplementary Methods 5
Workflow for the re-analysis of the pediatric SLE dataset 5
Calculation of silhouette width 5
Supplementary Tables 6
Supplementary Table S1 6
Supplementary Figures 7
Supplementary Figure S1 7
Supplementary Figure S2 8
Supplementary Figure S3 8
Supplementary Figure S4 9
Supplementary Figure S5 9
Supplementary Figure S6 10
Supplementary Figure S7 10
Supplementary Figure S8 11
Supplementary Figure S9 11
Supplementary Figure S10 12
Supplementary Figure S11 12
References 13

Supplementary Results

Comparing Ragas with existing subclustering integration methods

Although subclustering analysis has gained increasing popularity in single-cell research, it is mostly performed in an *ad hoc* manner without integrating subclusters from multiple cell compartments. In such “un-integrated” workflow, subclusters from different cell subsets are usually analyzed independently without the crosstalk between data objects from different subsets. Therefore, normalization and frequency analysis of subclusters, e.g., CD4+ naïve T cells in a PBMC study, are arbitrarily performed with respect to different levels of “total cells” by user’s choice, such as “total lymphocytes” (Perez, et al., 2022), “total T cells” (Stephenson, et al., 2021), or “total CD4+ T cells” (Terekhova, et al., 2023), leading to partial or even biased representation of phenotypic changes in the data.

A naïve approach to integrate subclusters is to simply “relabel” the cells with the newly-derived subcluster identities (Leary, et al., 2023; Zhu, et al., 2023). This would allow updating the refined cluster labels for rare subpopulations within the total cells. However, the relabeling-based method only updates the cell identities, not the cell embeddings, which often leads to poor separation of homogeneous subpopulations on a dimensional reduction plot, such as UMAPs.

As an improvement to the relabeling-based integration method, Leary et al., recently introduced a tool called SCISSORS for semi-supervised reclustering analysis (Leary, et al., 2023). To the best of our knowledge, SCISSORS is the only computational tool that supports multi-level re/subclustering analysis, which evaluates the goodness-of-fit of existing clusters using silhouette scores and hence suggests poorly-fit clusters for further reclustering. Same as the abovementioned relabeling method, one key limitation of SCISSORS is that it can only “integrate” subclustering results by re-assigning subcluster labels at the total cell level, which lacks the ability to enhance the visualization of rare or homogeneous subpopulations. Another limitation of the SCISSORS framework is its limited flexibility and compatibility with key scRNA-Seq preprocessing steps. For instance, SCISSORS only supports batch-integration using Seurat’s anchor-based method.

As a post-integration framework for multi-level subclustering analysis, Ragas has the following major advantages compared to existing subclustering practices (Table S1):

(1) Unlike existing practices that superficially integrate subclusters by re-label cells, Ragas is the first method to integrate cell embeddings from multi-level subclustering analysis by combining KNNs, which significantly improves the visualization of rare and homogeneous subpopulations (Table S1.I-II).

(2) As an extension to the Seurat data structure, the Pi object implemented in Ragas stores subcluster analysis results and seamless connects analytical data with visualization functions (Table S1.III).

(3) The Pi object can crosslink subcluster objects from multiple levels, allowing users to comprehensively analyze and compare cell frequency changes within different cell compartments (Table S1.IV).

(4) Ragas provides a highly flexible interface that is compatible with popular single-cell pre-processing and analysis workflows (Table S1.V). See vignette

https://github.com/jig4003/Ragas/blob/main/vignettes/CrossCompatibilityExamples.md for more details.

The re-projection weight parameter w

The re-projection weight parameter w, which is controlled by the "rp.weight" argument of the "CreatePostIntegrationObject" function, determines the trade-off between local subcluster separability and the global cluster connectivity. It takes value between 0 and 1 (default), with w=1 being the most conservative in preserving the original nearest neighbor structures before the re-clustering, while w=0 leads to the maximum separability between the new subclusters. In practice, we find that the default w=1 is a quite robust parameter that leads to very good balance between local separability and global connectivity. In some case, users may want to highlight the expression of some rare/homogeneous subpopulations, this is when w < 1 can be applied.

Fig. S1 gives an example showing re-projection of memory CD4+ T cells and Treg subsets to total T cells from the pediatric SLE dataset. Three different w values were used, including 1, 0.5, and 0. Based on Fig. S1, we can see a poor separation of CD4+ memory subsets on the original total T cell UMAP before subclustering (Fig. S1a). The separation gets significantly improved after re-projection with w=1 (Fig. S1b). As we decrease the value of w, CD4+ memory and regulatory T cells get further untangled from each other, and also from the rest of the T cell clusters (Fig. S1c-d). When w is set to 0.5, the re-projected UMAP robustly maintains a good a balance between local subcluster separability and global connectivity (Fig. S1c).

When w is set to 0, over-separation may occur. For instance, the Treg subclusters in Fig. S1d seems a little disconnected from the rest of the CD4+ T cells. In this case, one may not want to use such extreme w value if the goal is to visualize Treg subclusters at the total T cell level. However, this does not necessarily mean that the "isolated" subclusters from small w are always unfavorable outcomes of the re-projection algorithm. For instance, if we continue to integrate the re-projected (w=0) T cell object with the total PBMC object, both the Treg and the memory CD4+ subclusters will be in close proximity to the CD4+ T cells, including naïve CD4+ T cells and ISG-high T cells, resulting in a faithful representation of the cell lineages within the lymphocyte compartment (Fig. S2).

To quantitatively evaluate subcluster separability before and after re-projection, and comparing among different w values, we calculated the cell-level silhouette width as a measure of the agreement between the distribution of cells on the UMAP and their cluster assignment (Fig. S3). It is evident that as w decreased, many re-clustered subsets (e.g., CD4 TCM, Th17, Th22, naïve/memory Tregs, etc.) had increased silhouette scores, indicating improved local representation of T cell subcluster identities on total T cells. Interestingly, clusters without undergoing subclustering but were adjacent to CD4+ memory T cells or Tregs, such as the ISG-high cluster, also benefited from decreased w as their boundaries with Tregs became more apparent. Finally, regardless the choice of w, the rest of the clusters without going through subclustering remained similar silhouette scores in the new UMAP.

In Summary, the parameter 'w' controls the trade-off between global connectivity and local subcluster separability for re-projection analysis. In a multi-level subclustering setting, here are a few guidelines for selection of w:

(1) The default w=1 is a robust choice for general subcluster integration.

(2) If better local separability is desired, one can decrease w to increase the separability of subclusters and with their neighboring clusters.

(3) In a multi-level subclustering setting, larger w might be preferred to avoid over-separation of subclusters at the top-level analysis (e.g., total PBMCs). For subclustering at lower levels (e.g., B or T cell subclusters from PBMCs), users are encouraged to try different w values since visually disconnected subclusters at lower levels may be glued back together to their corresponding neighboring cells when being integrated at a higher level.

Supplementary Methods

Workflow for the re-analysis of the pediatric SLE dataset

The Seurat guided-clustering workflow (https://satijalab.org/seurat/archive/v3.2/pbmc3k_tutorial) was followed to process scRNA-Seq data from the PBMCs of 33 pediatric SLE patients and 11 matched healthy donors, after removing doublets detected by Scrublet (Wolock, et al., 2019). Cells with percentage of mitochondrial expression greater than 10% and/or nFeature_RNA smaller than 700 or greater than 2700 were filtered. Sample level batch integration was performed using the Harmony (Korsunsky, et al., 2019) algorithm and the Harmony-corrected cell embeddings were further used for clustering (Blondel, et al., 2008) and UMAP analysis (McInnes, et al., 2018). After further removal of a small T-monocyte doublet clusters, a platelet cluster and erythrocyte cluster, we obtained a final cleaned PBMC dataset that was clustered and annotated with 15 initial cell types.

To enhance the resolution of lymphocyte subpopulations, we further performed multi-level subclustering analysis following the workflow described by Fig. 1a in the Main Text. We first subset total B and T cells and performed re-clustering on each compartment independently, followed by a second round of subclustering of CD4+ memory T cells and Tregs. For the subclustering analysis, we re-performed most of the pre-processing steps according to the abovementioned guided-clustering workflow, including variable feature selection, dimension reduction, before re-ran Harmony for batch integration. Finally, all subclusters from the multi-level subclustering analysis were sequentially re-projected back to the PBMC data (see vignette https://github.com/jig4003/Ragas/blob/main/vignettes/subcluster.md for more details).

Calculation of silhouette width

To measure the agreement between subcluster identities and cell distribution on the UMAP either before or after re-projection, silhouette width (Rousseeuw, 1987) was calculated using the silhouette function from the R package cluster. For computational considerations, random down-sampling was performed on the PBMC data (50,000 cell, Fig. 1, Main Text) and total T cell data (10,000 cells, Supplementary Fig. S3) before calculating the silhouette width.

Supplementary Tables

Supplementary Table S1

Table S1 Summary of the advantages of Ragas in subcluster analysis and integration compared to existing approaches.

	Un-integrated	Relabeling-based integration	SCISSORS	Ragas
I. Integrate subcluster identities	No	Yes	Yes	Yes
II. Re-project cells to improve subpopulation visualization	No	No	No	Yes
III. Specialized structure for subcluster data storage and visualization	No	No	No	Yes
IV. Integrate analysis across different subcluster levels	Manual	Manual	Manual	Automated
V. Flexibility and compatibility with existing scRNA-Seq processing tools	Highest	Highest	Limited	High

Supplementary Figures

Supplementary Figure S1

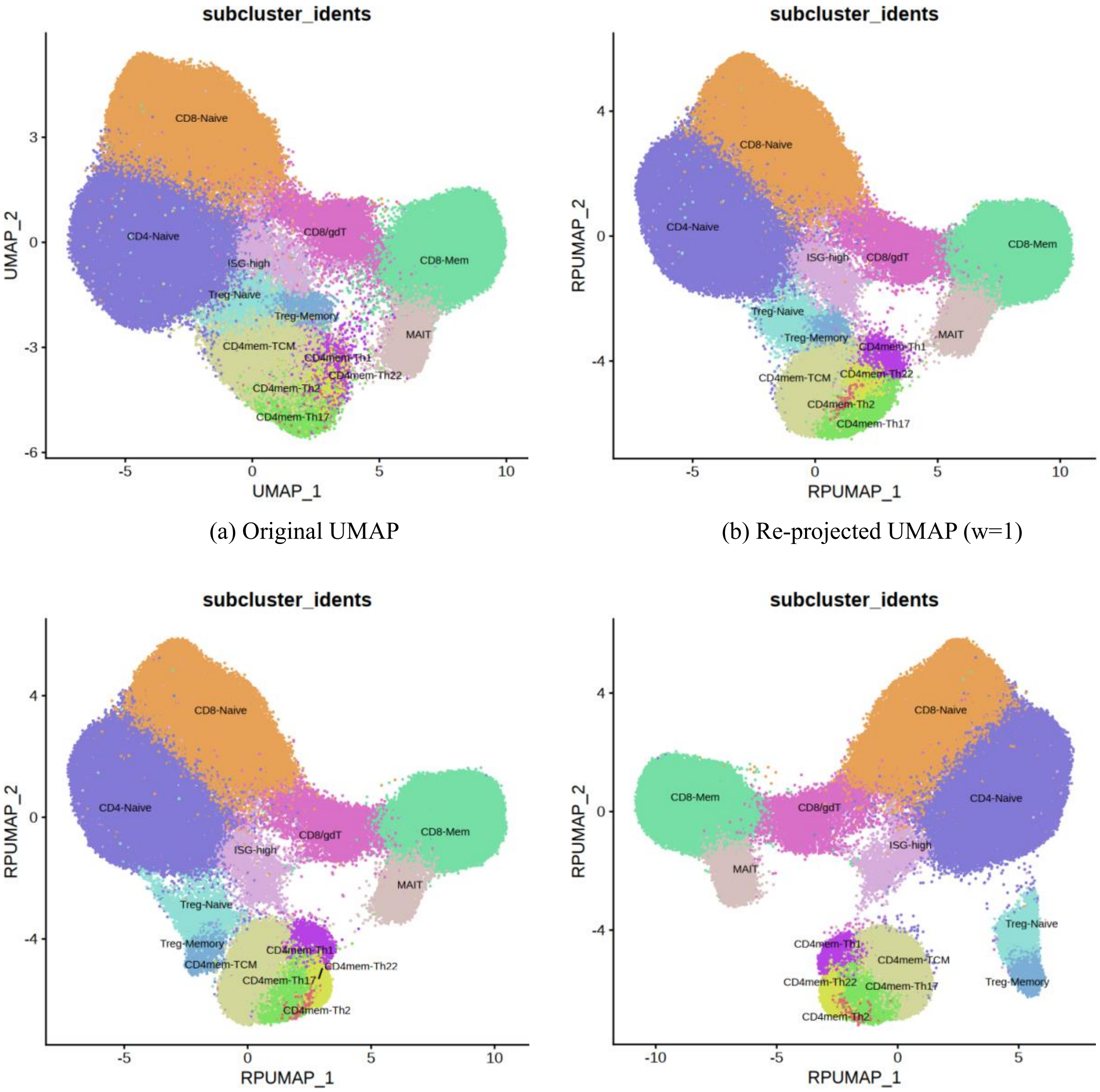

(a) Original UMAP

(b) Re-projected UMAP (w=1)

(c) Re-projected UMAP (w=0.5)

(d) Re-projected UMAP (w=0)

Fig. S1 UMAPs of total T cells from the pediatric SLE dataset before and after subcluster re-projection with different w values (w=0, 0.5, and 1).

Supplementary Figure S2

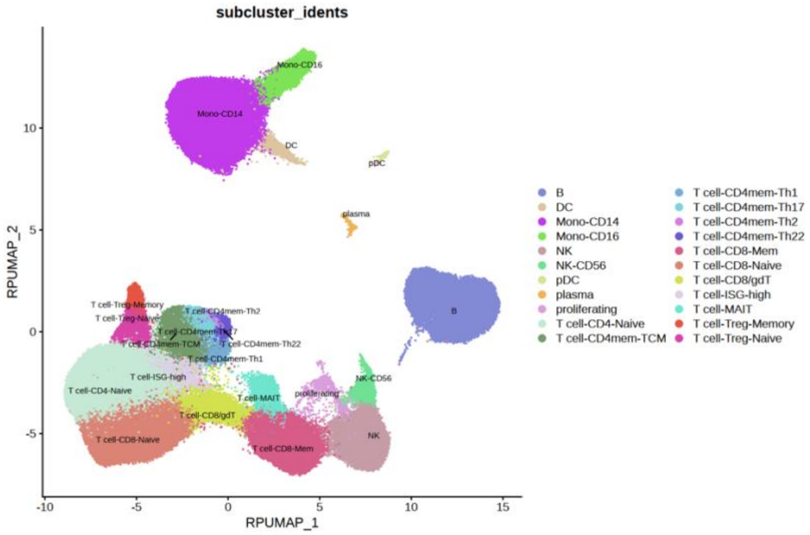

Fig. S2 UMAP showing the re-projected T cell object further integrated with the rest of PBMCs.

Supplementary Figure S3

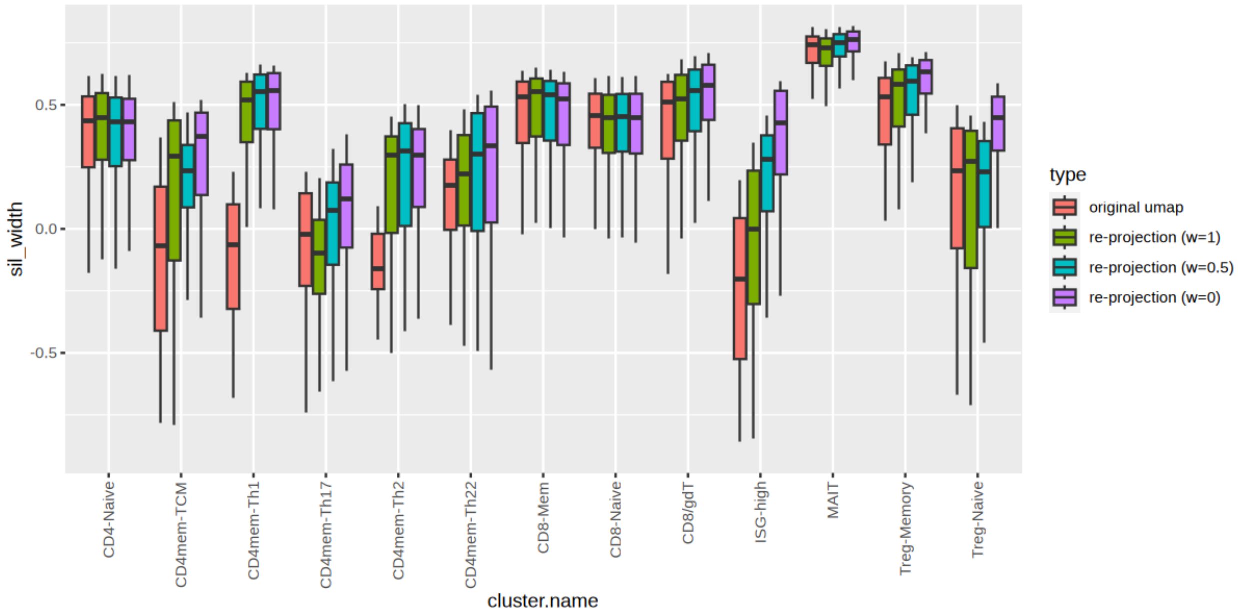

Fig. S3 Boxplot of the silhouette width distribution per subcluster for the original UMAP, and re-projected UMAP with w=0, 0.5, and 1.

Supplementary Figure S4

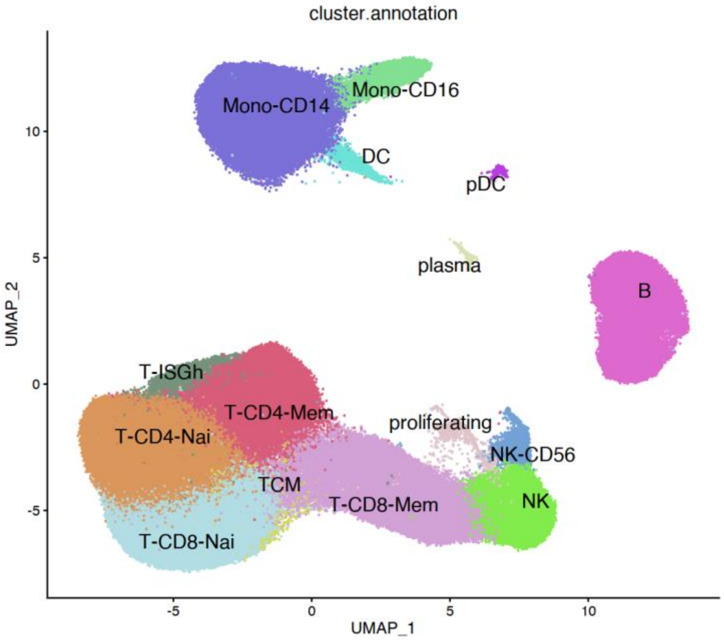

Fig. S4 UMAP of the pediatric SLE dataset from the initial main cluster analysis. 15 initial clusters are defined on total PBMCs from the main cluster analysis.

Supplementary Figure S5

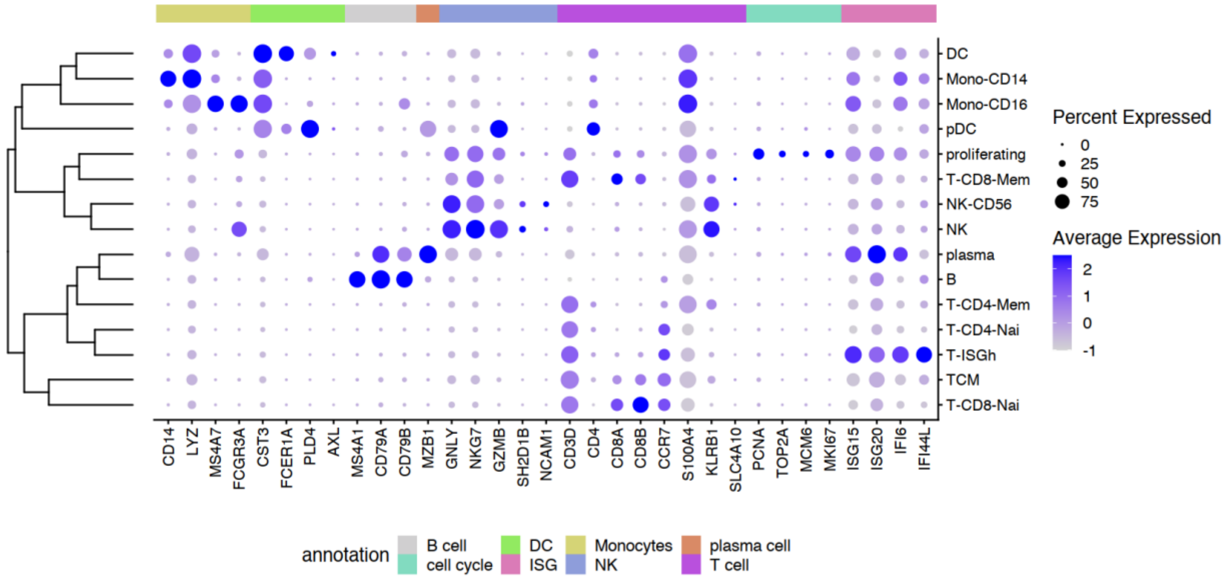

Fig. S5 Annotated Dot plot showing markers of major immune populations on total PBMC data.

Supplementary Figure S6

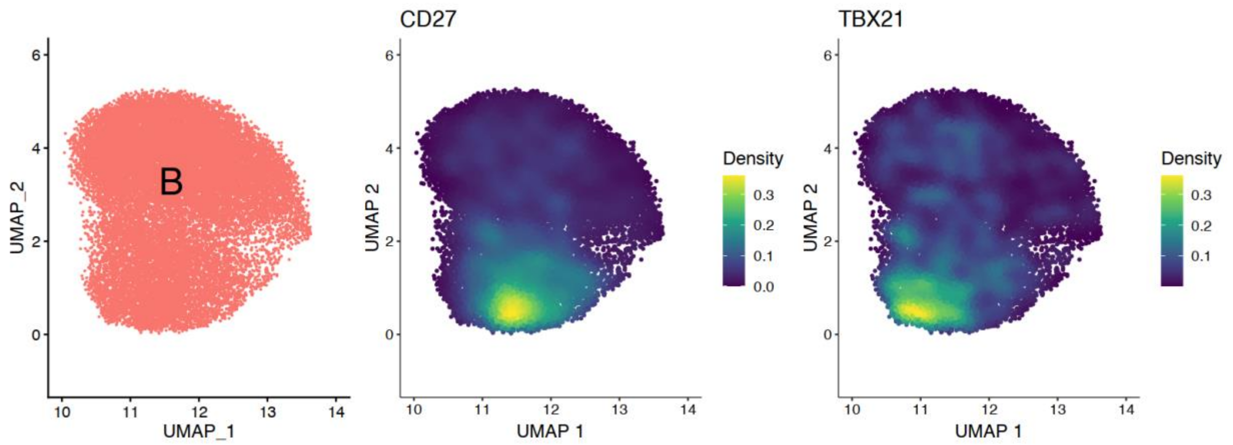

Fig. S6 Expression density plot for B cell subpopulations markers on B cell UMAP from the initial main cluster analysis. Markers for memory B cells (CD27) and DN2 (TBX21) largely overlap on the UMAP from main cluster analysis.

Supplementary Figure S7

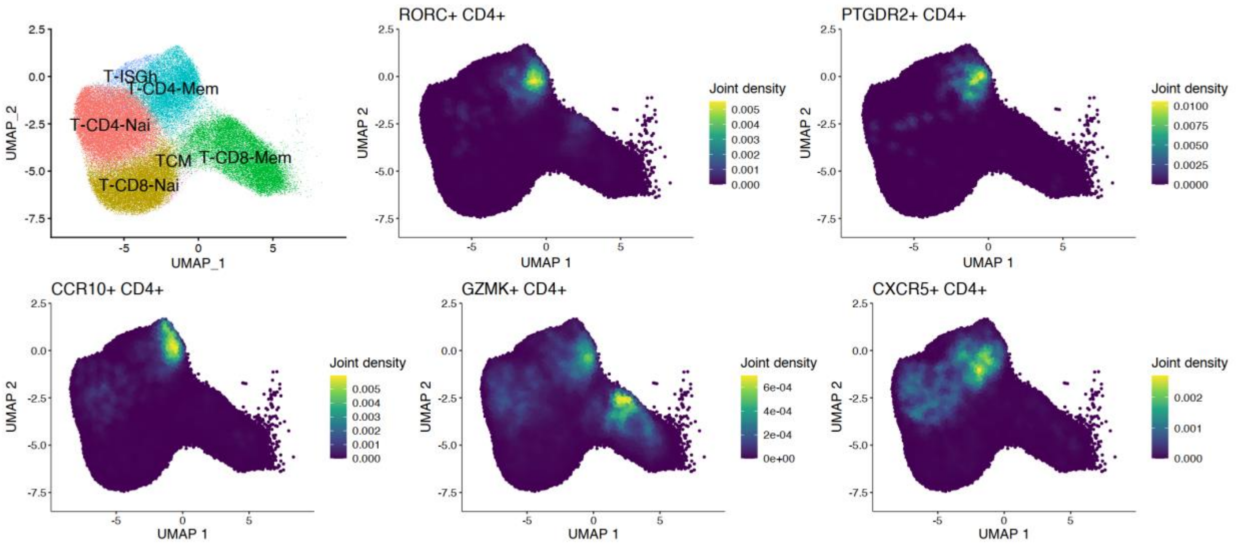

Fig. S7 Expression density plot for T helper cell subpopulations markers on T cell UMAP from the initial main cluster analysis. Markers for CD4+ T helper cells, including Th1 (GZMK), Th2 (PTGDR2), Th17 (RORC), Th22 (CCR10), and TCMs containing blood Tfhs (CXCR5) visualized on the T cell UMAP from main cluster analysis. Cells expressing Th1, Th2, Th17 or Th22 markers largely overlap with one another on the UMAP. A portion of CD4+GZMK+ are clustered with CD8+ T cells.

Supplementary Figure S8

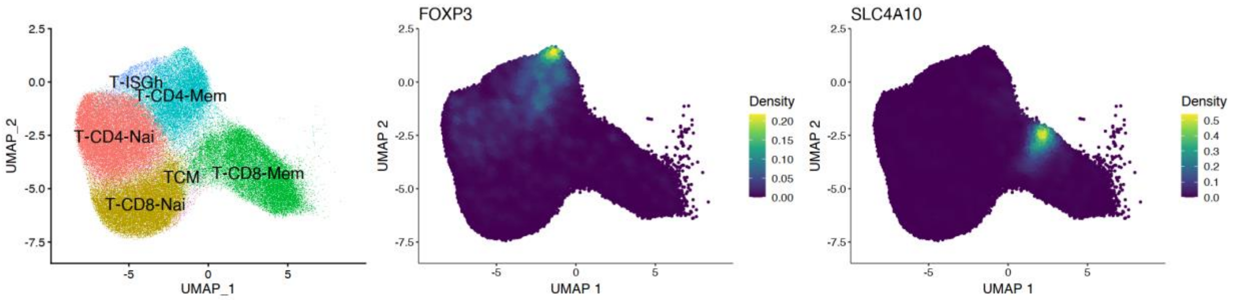

Fig. S8 Expression density plot for Tregs and MAIT cell markers on T cell UMAP from the initial main cluster analysis. Expression of Treg marker FOXP3 is distributed across memory CD4 cluster in the initial UMAP. MAIT cells expressing SLC4A10 are mixed in with CD8+ T cells.

Supplementary Figure S9

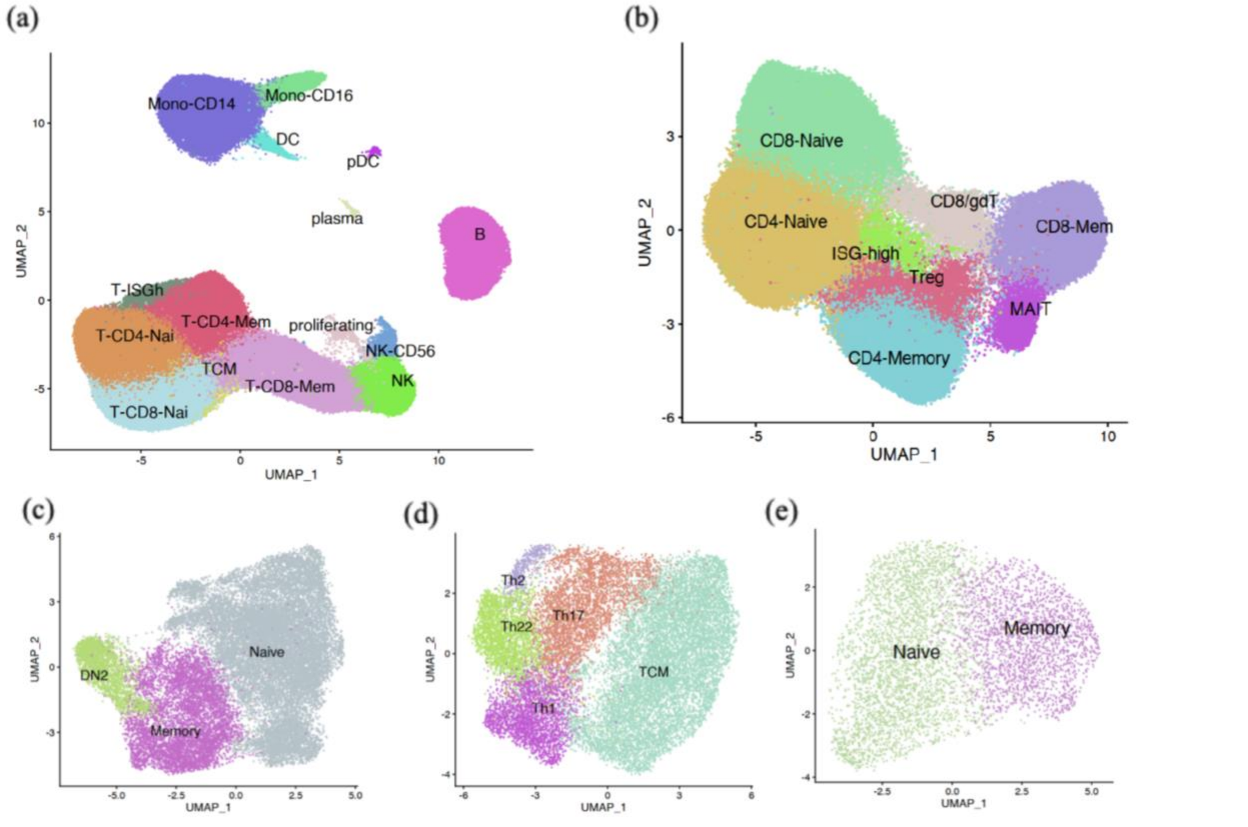

Fig. S9 Two-level subcluster analysis of single-cell PBMC data. (a) The initial UMAP describing 15 clusters in total PBMCs from main cluster analysis. (b-c) The 1st subcluster analysis of total T cells (b) and B cells (c). (d-e) The 2nd subcluster analysis focused on CD4+ memory T cells (d) and Tregs (e).

Supplementary Figure S10

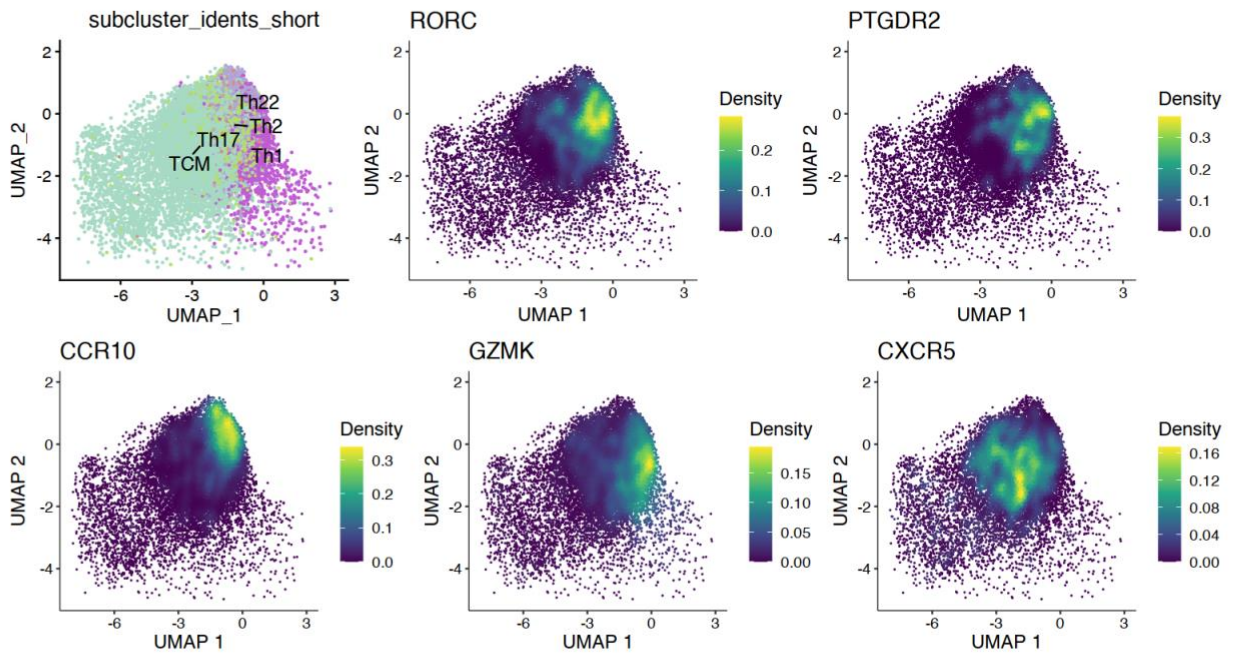

Fig. S10 Expression density plot for T helper cell subpopulations markers on CD4+ memory T cell UMAP from the initial main cluster analysis. Cells from different T helper subsets mixed together on the UMAP. Markers for Th2 (PTGDR2), Th17 (RORC), and Th22 (CCR10) cells largely overlap with one another.

Supplementary Figure S11

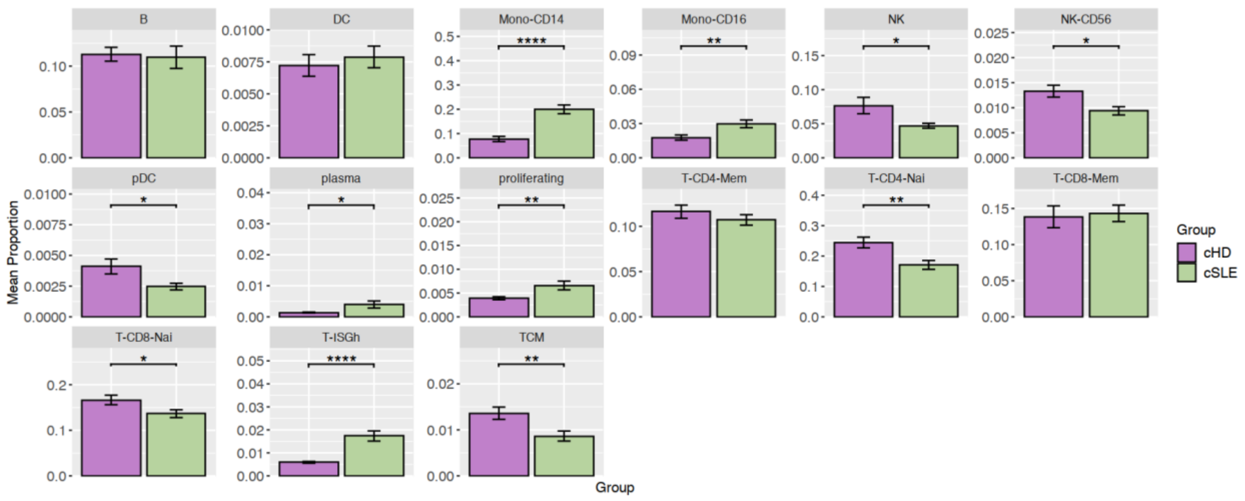

Fig. S11 Differential cell proportion analysis between SLE and healthy on the 15 main clusters in Supplementary Fig. S1. T cell clusters, including naïve CD4+ and CD8+ T cells, TCM, and memory CD4+ T cells, all show reduced frequency in children with SLE compared to healthy children.

References

Blondel, V.D., *et al.* Fast unfolding of communities in large networks. *Journal of Statistical Mechanics: Theory and Experiment* 2008;2008(10):P10008.
Korsunsky, I., *et al.* Fast, sensitive and accurate integration of single-cell data with Harmony. *Nat Methods* 2019;16(12):1289-1296.
Leary, J.R., *et al.* Sub-Cluster Identification through Semi-Supervised Optimization of Rare-Cell Silhouettes (SCISSORS) in single-cell RNA-sequencing. *Bioinformatics* 2023;39(8).
McInnes, L., *et al.* UMAP: Uniform Manifold Approximation and Projection. *The Journal of Open Source Software* 2018;3(29):861.
Perez, R.K., *et al.* Single-cell RNA-seq reveals cell type-specific molecular and genetic associations to lupus. *Science* 2022;376(6589):eabf1970.
Rousseeuw, P.J. Silhouettes: A graphical aid to the interpretation and validation of cluster analysis. *Journal of Computational and Applied Mathematics* 1987;20:53-65.
Stephenson, E., *et al.* Single-cell multi-omics analysis of the immune response in COVID-19. *Nat Med* 2021;27(5):904-916.
Terekhova, M., *et al.* Single-cell atlas of healthy human blood unveils age-related loss of NKG2C(+)GZMB(-)CD8(+) memory T cells and accumulation of type 2 memory T cells. *Immunity* 2023;56(12):2836-2854 e2839.
Wolock, S.L., Lopez, R. and Klein, A.M. Scrublet: Computational Identification of Cell Doublets in Single-Cell Transcriptomic Data. *Cell Syst* 2019;8(4):281-291 e289.
Zhu, H., *et al.* Human PBMC scRNA-seq-based aging clocks reveal ribosome to inflammation balance as a single-cell aging hallmark and super longevity. *Sci Adv* 2023;9(26):eabq7599.
